# Sweet and Fat Taste Perception: Impact on Dietary Intake in Diabetic Pregnant Women—A Cross-Sectional Observational Study

**DOI:** 10.3390/nu17152515

**Published:** 2025-07-31

**Authors:** Inchirah Karmous, Rym Ben Othman, Ismail Dergaa, Halil İbrahim Ceylan, Cyrine Bey, Wissem Dhahbi, Amira Sayed Khan, Henda Jamoussi, Raul Ioan Muntean, Naim Akhtar Khan

**Affiliations:** 1 Research Unit on Obesity UR18ES01, Faculty of Medicine, University of Tunis El Manar, Tunis 1007, Tunisia; inchirah.karmous@yahoo.fr; 2 Faculty of Medicine of Tunis, University of Tunis El Manar, Tunis 1007, Tunisia; benothmanr@gmail.com; 3 National Institute of Nutrition and Food Technology, Tunis 1007, Tunisia; hendajamoussi@gmail.com; 4 Higher Institute of Sport and Physical Education of Ksar Saïd, University of Manouba, Manouba 2010, Tunisia; phd.dergaa@gmail.com; 5 Physical Education of Sports Teaching Department, Faculty of Sports Sciences, Atatürk University, Erzurum 25030, Türkiye; 6 Faculty of Medicine of Sousse, University of Sousse, Sousse 4002, Tunisia; cyrine.b98@gmail.com; 7 Research Unit “Sport Sciences, Health and Movement”, High Institute of Sports and Physical Education of Kef, University of Jendouba, Kef 7100, Tunisia; wissem.dhahbi@gmail.com; 8 Training Department, Police College, Qatar Police Academy, Doha 7157, Qatar; 9 Institut National de la Santé et de la Recherche Médicale Research Center U1231, Team Physiology of Nutrition & Toxicology, Faculty of Life Sciences, University Bourgogne Franche-Comté, 21000 Dijon, France; amira.khan@u-bourgogne.fr (A.S.K.); naim-akhtar.khan01@u-bourgogne.fr (N.A.K.); 10 Department of Physical Education and Sport, Faculty of Law and Social Sciences, University “1 Decembrie 1918” of Alba Iulia, 510009 Alba Iulia, Romania

**Keywords:** gestational diabetes, taste perception, dietary intake, linoleic acid, maternal health

## Abstract

**Background**: Taste changes are common during pregnancy and can have a significant impact on dietary habits. **Objective**: This study aimed to investigate the influence of the perception of sweet and fat taste on diet in pregnant diabetic women. **Methods**: This cross-sectional observational study included 66 pregnant women, 33 with gestational diabetes and 33 with pre-gestational type 2 diabetes. Taste perception tests were conducted to evaluate thresholds for detecting sweet and fatty tastes. Dietary surveys were used to assess daily nutrient intake, and various biochemical parameters, such as glycemia, HbA1c, and cholesterol, were analyzed. **Results**: The low-fat taster group (threshold > 0.75 mmol/L) included more patients with diabetes compared to those with gestational diabetes. All diabetic patients had low sucrose perception. Although pregnant women with gestational diabetes detected sweetness at high concentrations, pregnant women with diabetes detected it at lower concentrations (0.012 ± 0.023 mmol/L vs. 0.006 ± 0.005 mmol/L; *p* = 0.3). High-fat tasters exhibited elevated glycemia compared to low-fat tasters (6.04 ± 1.88 mmol/L vs. 7.47 ± 3.4 mmol/L; *p* = 0.03). They also had higher cholesterol (*p* = 0.04) and lower HDL-C levels (4.96 ± 1.04 mmol/L vs. 1.36 ± 0.29 mmol/L; *p* = 0.03). High-fat tasters showed more frequent daily consumption of oil, butter, cheese, and chocolate. The highly sweet tasters had higher cholesterol levels and lower LDL levels. Individuals who reported being highly sensitive to sweet taste consumed more daily oil, sweetened yogurt, or cream desserts, as well as white sugar. **Conclusions**: These findings indicate that altered sensitivity to fat and sweet tastes is associated with different dietary habits and metabolic profiles in pregnant women with diabetes. Specifically, reduced sensitivity to the taste of fat is associated with higher consumption of high-fat foods and poorer lipid profiles. In contrast, sensitivity to sweet taste correlates with an increased intake of sugary and fatty foods. Understanding these taste-related behaviors can help develop personalized nutritional strategies to improve metabolic control and maternal–fetal outcomes in this high-risk group.

## 1. Introduction

About 422 million people worldwide have diabetes; the majority are in low-and middle-income countries, and 1.6 million deaths are directly attributed to diabetes each year. Both the number of cases and the prevalence of diabetes have been steadily increasing in recent decades, including Tunisia [1].

The most common types of diabetes are type 1 diabetes, type 2 diabetes, and gestational diabetes. According to the World Health Organization (WHO), gestational diabetes is defined as a disorder of glucose tolerance leading to hyperglycemia of varying severity, first diagnosed during pregnancy, regardless of the necessary treatment and the course in the postpartum period [1]. In Tunisia, the proportion of patients with gestational diabetes has increased in 10 years from 21.7% to 72.5% of all women hospitalized for diabetes and pregnancy [2].

During pregnancy, there is an increase in metabolic needs to meet the requirements of both the mother and the fetus, resulting in increased food intake. This change in food intake can be both quantitative and qualitative in nature. Abnormal consumption and an increased preference for foods rich in lipids and sugars are detected in pregnant women with gestational diabetes and in pregnant women who are already diabetic [3,4,5]. Gustatory function was reduced during pregnancy compared to non-pregnant women, leading to dietary changes in some pregnant women [6].

Historically, researchers believed that the texture and smell of lipids were primarily responsible for the increase in attraction to foods high in fat. However, recent studies have shown that taste plays a key role in fat preference [6,7]. Several taste modalities are identified: bitter, sweet, umami, sour, salty, and fatty. Perception of these tastes varies from person to person, and researchers have suggested that taste perception may influence eating habits and nutritional status, potentially contributing to the development of metabolic diseases, as seen in gestational diabetes [8].

Despite these findings, there is a lack of research on how taste perception influences food intake in pregnant women with diabetes. Most studies have focused on the general population or non-pregnant individuals, leaving a gap in understanding the particular nutritional issues of pregnant women with diabetes. Addressing these gaps is crucial for developing targeted nutritional interventions that can help manage diabetes and improve maternal and fetal health.

Therefore, our study aimed to (i) evaluate the threshold for perception of fat taste and sweet taste in both groups of women, and (ii) assess the relationship between taste sensibility and dietary survey and biological parameters in pregnant women with gestational diabetes compared to diabetic pregnant women.

## 2. Materials and Methods

### 2.1. Ethical Approval

This study was conducted in accordance with the guidelines of the Declaration of Helsinki and was approved by the ethical committee of the Tunisian National Institute of Nutrition and Food Technology (approval number: 03/2021, dated 1 March 2021). All study participants provided their informed consent.

### 2.2. Sample Size

We used G.Power version 3.1.9.7 to calculate the sample size with a *t*-test for independent variables. The effect size was fixed at 0.3, with a significance level of α = 0.05 and (1-β) = 0.8. The total sample size was 64.

### 2.3. Participants

A cross-sectional observational study was performed on 66 pregnant women with gestational diabetes (*n* = 33) and pre-gestational type 2 diabetes (*n* = 33) from the outpatient department of the National Institute of Nutrition (Tunis, Tunisia). The recruitment was conducted between September and December 2019.

**Figure 1:**
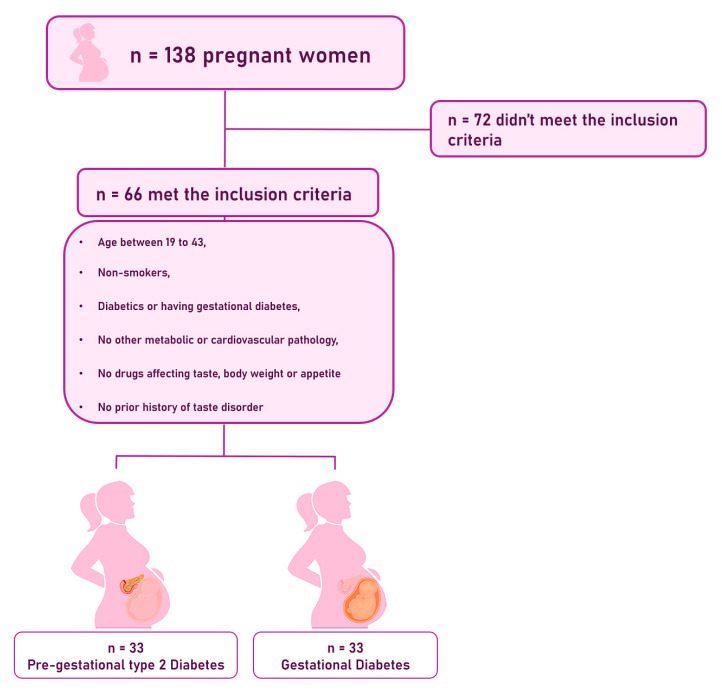
Flowchart of the population.

The inclusion criteria were pregnant women aged 19 to 43 years, non-smokers, diabetics or having gestational diabetes, without any other metabolic or cardiovascular pathology, without taking drugs that affect taste, body weight, or appetite, and without a history of taste disorder. Figure 1 illustrates the flowchart of the population.

### 2.4. Study Procedure

All pregnant women were recruited at their first visit to the Outpatient Department. The diagnosis of gestational diabetes was performed by a nutritionist doctor according to the American diabetes association criteria [9]. The diagnosis of pre-gestational type 2 diabetes was noted from the file of the patients. A questionnaire was administered, followed by a 24-hour recall and biochemical analysis. If patients were not fasting, they were asked to return in one week to undergo the lab tests. After that, pregnant women were assessed for their perception of sweet and fatty tastes, as described below.

### 2.5. Methods and Measurements

#### 2.5.1. Blood Sample Collection and Biochemical Analysis

Blood samples were collected from all participants, and blood analysis was performed in the biochemistry laboratory at the National Institute of Nutrition in Tunis, Tunisia.

This assessment includes glycemia, glycated hemoglobin HbA1c, and a lipid assessment (triglycerides, total cholesterol, high-density lipoproteins HDL, and low-density lipoproteins LDL).

#### 2.5.2. Tasting Tests

To evaluate oral sensitivity to sweet and fat tastes, we used sucrose to assess sweet taste perception and linoleic acid to assess sensitivity to fat taste [10]. All samples were collected at the same time of day to minimize the effect of time of day on the accessed parameters [11]. All participants must be fasting, having not eaten anything in the two hours preceding the test. All taste stimuli were prepared on the day before use and stored at 5 °C. Taste detections were selected based on values commonly reported in the literature as representative of human sensory detection limits. These concentrations reflect the minimum levels at which taste receptors are typically activated and participants begin to reliably perceive the respective taste qualities in controlled settings [12]. All taste tests were conducted blind to participants’ diabetes status to minimize potential bias.

#### 2.5.3. Linoleic Acid Sensitivity Analysis

Fat taste was assessed by employing linoleic acid at different ascending concentrations (0.018, 0.18, 0.37, 0.75, 1.5, 3.6, and 12 mmol/L) by sip and spit technique and a three-alternative forced choice method [10].

Three solutions were prepared: two control solutions, each containing water and 5% arabic gum, and one test solution, which contained water, 5% arabic gum, and linoleic acid [10].

If the participant is unable to detect a difference in “taste”, they are asked to test the next strength solution until they can detect the presence of an oily taste to note the detection limit [10].

As soon as the patient reports detecting the taste corresponding to the tested solution, the response is validated positively. Then, based on the obtained results, we divided the population into two groups: low tasters and high tasters, with the low tasters having a threshold value of 0.75 mmol/L. The precise dilution methodology is described in the article by Karmous et al. [10].

#### 2.5.4. Sweet Taste Sensitivity Analysis

Taste preference tests for sweet taste were performed using sucrose at different ascending concentrations (0.64, 0.32, 0.16, 0.08, 0.04, 0.02, 0.01, 0.005, 0.0025, and 0.00125 mol/L) by the sip-and-spit technique and a forced-choice method with three alternatives [13,14].

Three solutions were prepared, two of which contained water, considered as control solutions, and the third contained water and sucrose, regarded as the test solution.

If the participant is unable to detect a difference in “taste”, they are asked to test the next strength solution until they can detect the presence of a sweet taste, noting the detection limit. As soon as the patient reports detecting the taste corresponding to the tested solution, the response is validated positively. The results obtained divided the population into two groups: low tasters and high tasters, based on a threshold of 0.04 mol/L, with the non-tasters classified among the low tasters [15].

#### 2.5.5. Nutritional Analyses

An experienced dietitian assisted with the dietary recall. The analysis of the data from the food survey was conducted using Nutrilog version 3.20, which estimates daily caloric intake, spontaneous intakes of macronutrients and micronutrients (including vitamins and minerals), and water intake.

## 3. Statistical Analysis

Statistical analyses were performed using SPSS version 15.0 software. A descriptive analysis was conducted for quantitative variables, expressed as mean ± standard deviation, and qualitative variables, presented as percentages. Averages were compared using the Student’s *t*-test, with the Mann–Whitney test employed in cases of non-compliance. For percentage comparisons, Pearson’s chi-square test was utilized, and in instances of test invalidity, Fisher’s exact bilateral test was applied. In situations where the variable did not follow a normal distribution, the median and interquartile range were used.

Generalized linear models were used to test the association between taste sensitivity (sweet/fat) and the tested parameter levels (continuous variable), adjusting for confounding factors. We used binary logistic regression to test the association between taste sensitivity (sweet/fat) and categorical variables (e.g., type of diabetes). Adjustment was made for confounding factors. A significance level of *p* < 0.05 was considered statistically significant.

## 4. Results

### 4.1. Characteristics of the Population

Our population is composed of 66 diabetic pregnant women, 33 of whom had gestational diabetes, and 33 were diabetic before pregnancy. The mean age of the population was 30 ± 2 years.

For pregnant women with prior diabetes, the mean duration of diabetes was 6 ± 4 years. The average gestational age was 20 ± 8 weeks of amenorrhea. Glycated hemoglobin levels were significantly higher in pregnant women with diabetes than in women with gestational diabetes.

### 4.2. Fat Taste

Table 1 presents the characteristics of the population according to their preference for fat taste. Participants with low fat taste sensitivity had significantly higher rates of diabetes before pregnancy (84.4% vs. 17.6%; *p* < 0.001), lower taste thresholds for linoleic acid (0.75 [0.18–0.75] vs. 3.00 [1.50–6.00] mmol/L; *p* < 0.001), higher fasting glycemia (7.47 ± 3.40 vs. 6.05 ± 1.88 mmol/L; *p* = 0.042), lower HDL-C levels (1.35 ± 0.24 vs. 1.53 ± 0.30 mmol/L; *p* = 0.009), and higher magnesium intake (224 ± 86.2 vs. 176 ± 83.4 mg/d; *p* = 0.024) compared to high-fat tasters. Total cholesterol was significantly lower among low-fat tasters in both unadjusted (*p* = 0.027) and adjusted (*p* < 0.001) comparisons. After adjusting for confounding variables (type of diabetes, age, BMI, cholesterol, and HbA1c), significant differences remained for diabetes before pregnancy (*p* < 0.001), linoleic acid taste threshold (*p* < 0.001), HbA1c (7.05 ± 1.67 vs. 6.28 ± 1.51%; *p* = 0.012), and triglyceride levels (1.32 [1.05–1.71] vs. 1.55 [1.04–1.99] mmol/L; *p* = 0.019).

Table 2 represents the characteristics of the population categorized by sweet taste. Participants with low sweet taste sensitivity had significantly lower rates of gestational diabetes compared to those with high sweet taste sensitivity (15.6% vs. 82.4%; *p* < 0.001). They also exhibited lower taste thresholds for sucrose (0 vs. 0.50 [0.25–1.00] mmol/L; *p* < 0.001), higher HbA1c levels (6.84 ± 1.73 vs. 5.82 ± 0.56%; *p* = 0.001), lower total cholesterol (5.04 ± 1.03 vs. 6.15 ± 1.28 mmol/L; *p* = 0.002), and lower triglyceride levels (1.31 [1.02–1.69] vs. 1.75 [1.57–2.32] mmol/L; *p* = 0.016) compared to high-sweet tasters. After adjusting for potential confounders including type of diabetes, age, BMI, cholesterol, and HbA1c, significant differences remained for sucrose taste thresholds (*p* < 0.001), dietary fiber intake (22.3 ± 8.34 vs. 25.6 ± 8.13 g/day; *p* = 0.004), and vitamin C intake (91.0 [47.0–144] vs. 141 [61.0–230] mg/day; *p* = 0.004).

**Figure 2:**
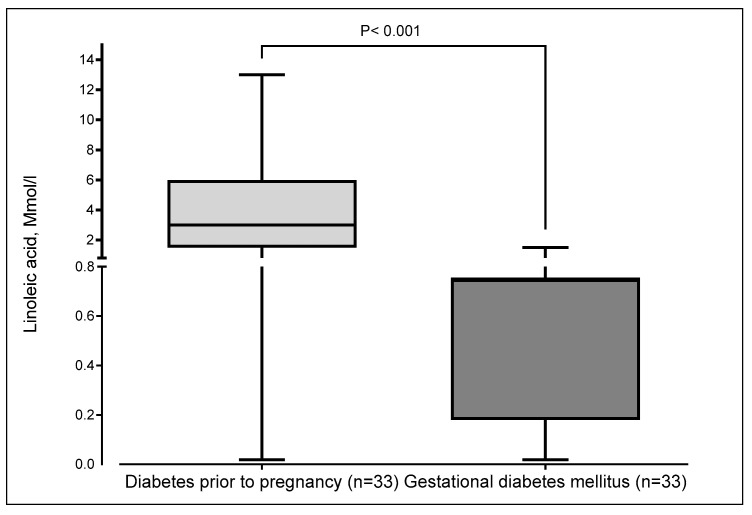
Thresholds of linoleic acid taste according to the type of diabetes in pregnant women. The boxplot displays minimum, first quartile (Q1), median, third quartile (Q3), and maximum. The median and the third quartile were superimposed in gestational diabetes mellitus.

Figure 2 illustrates the taste thresholds for linoleic acid among pregnant women with gestational diabetes (GDM) and those with type 2 diabetes (T2D). Women with T2D exhibited significantly higher thresholds compared to those with GDM (*p* < 0.001), indicating reduced sensitivity to linoleic acid taste.

Among laboratory parameters, adjusted analyses revealed significant differences in HbA1c (*p* = 0.012), total cholesterol (*p* < 0.001), and triglycerides (*p* = 0.019), favoring better metabolic profiles in high-fat tasters. Dietary intake showed no significant differences in macronutrient composition, but unadjusted magnesium intake was higher in low-fat tasters (*p* = 0.024), though this was not significant after adjustment (*p* = 0.102).

### 4.3. Sweet Taste

**Figure 3:**
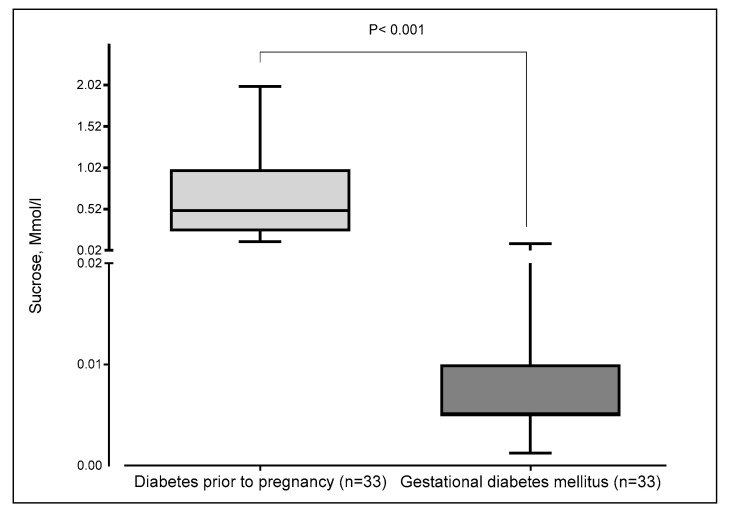
Thresholds of sucrose concentration according to the type of diabetes in pregnant women. The boxplot displays minimum, first quartile (Q1), median, third quartile (Q3), and maximum. The median and the first quartile were superimposed in gestational diabetes mellitus.

Women with pre-existing diabetes exhibited notably higher sucrose thresholds, indicating reduced sweet taste sensitivity, compared to women with GDM, whose thresholds were much lower. The difference is statistically significant (*p* < 0.001) (Figure 3).

## 5. Discussion

The primary objective of this study was to examine the impact of sweet and fatty taste perception on food intake in pregnant women with diabetes. Understanding these changes in taste perception is crucial for developing tailored nutritional interventions to improve dietary adherence and glycemic control in this population. This study found significant differences in taste perception and food intake in pregnant women with diabetes.

To the best of our knowledge, this is the first study worldwide to specifically investigate the combined effect of sweet and fat taste perception on food intake in pregnant women with diabetes. Previous research has primarily focused on changes in taste perception during pregnancy or in individuals with diabetes, without considering the unique challenges faced by pregnant individuals with diabetes. This study fills a critical gap in the literature by highlighting the importance of taste perception for nutritional management in this special population. Furthermore, this study is of particular significance as it is the first of its kind to be conducted in North Africa.

However, a major limitation is the absence of a non-diabetic pregnant control group. This limits the ability to determine whether the observed differences in taste perception and dietary preferences are specific to diabetes or are part of the regular physiological changes in pregnancy. Without this comparison group, it is difficult to isolate the effect of diabetes on taste perception from the broader effects of pregnancy itself.

### 5.1. Fat Taste Perception

The taste perception threshold for linoleic acid was significantly higher in pregnant women with prior diabetes than in pregnant women with gestational diabetes. In a study by Stewart et al., consumption of a high-fat diet significantly decreased taste sensitivity to long-chain fatty acids in healthy subjects. Additionally, the same study found that consumption of low-fat foods increased taste sensitivity in the study population. Hence, the authors suggest that a decrease in the ability to detect fat taste is a contributing factor to excessive consumption of foods rich in fat [16].

Recent studies have focused on CD36, a glycoprotein located at the lingual level, which acts as a fatty acid receptor. According to the literature, CD36 exhibits a broad spectrum of expression and plays a crucial role in the pathophysiology of diabetes, as well as in the dietary selection of fatty foods [13,14]. Recently, researchers detected soluble CD36 in the blood as an acellular form that participates in the absorption of fatty acids [17,18]. Thus, other researchers have found a very high serum CD36 level in newly diagnosed diabetic patients, comparing them to healthy subjects, and have suggested an association between this soluble form of CD36 and resistance to insulin [19]. Based on these results, it is recommended that the differences in orosensory fatty acid detection may be the result of lingual or serum CD36 polymorphism, which, in turn, is responsible for the increased preference for dietary lipids.

### 5.2. Sweet Taste Perception

Women with pre-existing diabetes had significantly higher sucrose detection thresholds, suggesting a lower sensitivity to sweet taste compared to those with gestational diabetes. Impairment in the taste for sucrose and similar sweet tastes has been well-documented in people with diabetes [20].

According to a study by Wasalathanthri et al., there was no change in sweet taste sensitivity in prediabetic subjects compared to those with regular blood sugar levels [21].

According to these results and the literature, hormones such as insulin and leptin can modulate sweet taste, and genetic variation is the cause of the difference in sweet taste detection. Indeed, Belzer et al. examined the associations between leptin and insulin, as well as the sensory evaluation of sweet taste. The results showed that in women with gestational diabetes, there was a strong correlation between the appreciation of 10% sucrose-flavored milk and the level of fasting serum leptin, as well as a strong correlation between the average sweet taste of sweet solutions and fasting serum insulin level [22].

Other studies have found that some people are more sensitive to sweet taste than others. According to these researchers, there is a relationship between the density of fungiform taste buds and the intensity of the sugar sensation: the denser the taste buds, the greater the intensity of the sweet taste sensation [23,24]. On the genetic level, Eny et al. showed in their research that a high sugar intake is associated with a genetic variation in TAS1R2. This gene encodes the receptor responsible for sweet taste in individuals with obesity [25]. The single-nucleotide polymorphism (SNP) rs12033832 in the TAS1R2 gene has been associated with lower sugar sensitivity and higher sugar intake in overweight individuals [26]. This suggests that genetic variations can influence how individuals perceive sweetness and their subsequent sugar consumption.

Although the perception of sweet taste is mediated by the T1R2/T1R3 receptors, which can recognize all chemical compounds perceived as sweet by humans, these receptors also have a newly identified role: regulating metabolic processes [27].

Hence, a relationship exists between sweet taste sensitivity and digestive hormones, such as leptin, and it is suggested that differences in sweet taste detection may be genetic. Indeed, Garcia-Bailo, in his study on gene-taste interaction, found that the genetic variability of the gene responsible for taste perception can be the cause of differences in preferences and eating habits between people [28]. Excessive consumption of fat or sugar can have a significant impact on the offspring, influencing their taste preferences and predisposing them to future metabolic diseases, as demonstrated in animal models by Mezei et al. [29].

### 5.3. Association of Lipid and Sugar Detection Thresholds with Biochemical Parameters in Diabetic Pregnant Women

The results of our study showed a positive correlation between hemoglobin and blood sugar levels, as well as long-chain fatty acid detection thresholds, and a negative correlation between the fat detection threshold and cholesterol and HDL-C levels. This observation aligns with prior research indicating that metabolic status can modulate taste perception. For instance, Stewart et al. [30] reported altered lipid taste sensitivity in individuals with obesity and insulin resistance, potentially due to changes in the expression of CD36, a fatty acid translocase involved in fat taste perception.

The negative correlation between fat detection thresholds and cholesterol and HDL-C levels suggests that individuals with better lipid profiles may have heightened sensitivity to fat. This is supported by findings from Newman et al. [31], who demonstrated that variations in orosensory fat detection thresholds are associated with blood lipid concentrations and may influence dietary fat intake and preferences. However, as in our study, the directionality and causality of these relationships remain uncertain and could be influenced by genetic, metabolic, or behavioral factors. While these correlations were statistically significant, it is essential to note that some of the observed differences—such as those in HbA1c and lipid levels—may be clinically marginal, suggesting limited immediate clinical relevance. As emphasized in the work of Drewnowski et al. [32], small shifts in biomarkers may not necessarily reflect meaningful changes in metabolic health, especially in cross-sectional designs. Therefore, these findings should be interpreted with caution.

For sweet taste, our results revealed no relationship between the sweet taste detection threshold and various biochemical parameters. Likewise, a study by Ji Hee Yu et al., on diabetic patients, showed that there is no significant association between the preference for sugary foods, specifically sucrose, and the values of fasting blood sugar and glycated hemoglobin [33]. This contrasts with some literature suggesting a link between glycemic control and sweet taste sensitivity, particularly in patients with type 2 diabetes [34]. However, other studies, such as those by Grüneis et al. [35], have reported inconsistent or null associations, which may reflect variability in methodology, sample characteristics, or the multifaceted nature of sweet taste perception, which involves central and peripheral mechanisms beyond metabolic parameters alone.

## 6. Limitation

This study has several limitations. Firstly, the sample size is relatively small, consisting of 66 pregnant women, with 33 having gestational diabetes and 33 with pre-existing diabetes. This may limit the generalizability of the findings to a broader population. This small sample size, particularly when performing subgroup comparisons, may also result in insufficient statistical power to detect subtle but potentially meaningful effects. Additionally, the recruitment period occurred between September and December 2019, which may have introduced seasonal variations in dietary habits that were not accounted for in this study. Another limitation is the reliance on perception tests and nutritional surveys, which are subjective and may be influenced by individual reporting biases. The lack of a control group without diabetes further restricts the ability to compare findings with non-diabetic pregnant women. Moreover, the cross-sectional nature of this study limits the ability to draw causal inferences. A longitudinal follow-up would strengthen the conclusions by allowing for assessment of changes over time and potential causal relationships. Furthermore, dietary intake data were collected using a single 24-hour recall, which introduces considerable day-to-day variability and limits the representativeness of usual dietary patterns.

## 7. Conclusions

This study offers valuable insights into the impact of gestational and pre-existing diabetes on taste sensitivity and dietary preferences in pregnant women. Our results show that pregnant women with diabetes show a marked preference for high-fat foods, especially those rich in saturated and trans fatty acids. This preference is associated with a higher threshold for the perception of linoleic acid, suggesting a lower sensitivity to fat tastes in pregnant women with diabetes compared to pregnant women with gestational diabetes. While this altered taste perception may contribute to the increased consumption of high-fat foods observed in this population, it is essential to note that the associations between taste sensitivity and lipid levels are likely multifactorial.

Interestingly, our study found that sensitivity to sweet taste was not affected by diabetes, as both groups of pregnant women had similar thresholds for perceiving the taste of sucrose. This suggests that the changes in taste perception due to diabetes are more pronounced for fat tastes than for sweet tastes. The importance of this study lies in its potential to inform nutritional interventions and public health strategies aimed at improving maternal and fetal health in pregnant women with diabetes. Understanding the specific changes in taste perception in this population is critical for developing tailored dietary recommendations that can enhance dietary adherence and metabolic control.

From a practical perspective, these results highlight the importance of personalized nutritional advice that takes into account individual taste preferences and sensitivities. For example, pregnant diabetics with a higher threshold for fat tastes could benefit from strategies that reduce the consumption of high-fat foods and promote healthier alternatives. This could mean that they consume more flavorful, low-fat foods that match their taste preferences without compromising their metabolic health. In addition, this study highlights the importance of monitoring and managing fat intake in pregnant diabetics to avoid excessive consumption of unhealthy fats, which can exacerbate metabolic complications. Incorporating a nutrigenetic approach—such as investigating the role of TAS1R2 polymorphisms in sweet taste perception and their relationship with dietary behavior and diabetes susceptibility—could further enhance the clinical relevance of our results and potentially improve patient compliance. Healthcare providers should consider these changes in taste perception when advising on food choices and creating personalized diet plans. In practice, this research can inform the development of educational programs and resources for pregnant women with diabetes, enabling them to make informed dietary choices that support both their health and the health of their developing fetuses. Future research should further investigate the underlying mechanisms of changes in taste perception and their impact on dietary behavior and metabolic health in pregnant women with diabetes.

## Figures and Tables

**Table 1:** Comparison of Demographic and Biochemical Parameters by Fat Taste Sensitivity.

		Low-Fat Tasters *n* = 32	High-Fat Tasters *n* = 34	*p*	*p* Adjusted
Diabetes before pregnancy (*n*)		27 (84.4)	6 (17.6)	<0.001	<0.001
Gestational diabetes (*n*)		5 (15.6)	28 (82.4)		
BMI (Kg/m^2^)		30.1 ± 6.36	30.9 ± 5.08	0.567	
Taste thresholds for linoleic acid (Mmol/L) ^¥¥^		0.75 (0.18–0.75)	3.00 (1.50–6.00)	<0.001	<0.001
Gestational age (month)		6.68 ± 1.85	5.72 ± 2.11	0.054	
Laboratory tests	Glycemia ^¥^ (mmol/L)	7.47 ± 3.40	6.05 ± 1.88	0.042	0.452
	HbA1c ^¥^ (%)	7.05 ± 1.67	6.28 ± 1.51	0.054	0.012
	Cholesterol ^¥^ (mmol/L)	4.92 ± 0.99	5.54 ± 1.22	0.027	<0.001
	HDL-C ^¥^ (mmol/L)	1.35 ± 0.24	1.53 ± 0.30	0.009	0.258
	LDL-C ^¥^ (mmol/L)	3.11 ± 0.81	3.29 ± 0.94	0.398	0.756
	Triglycerides ^¥^ (mmol/L)	1.32 (1.05–1.71)	1.55 (1.04–1.99)	0.261	0.019
Dietary survey	Calories ^¥¥^ (Kcal/d)	2527 ± 719	2712 ± 1059	0.411	0.096
	Carbohydrates ^¥¥^ (g/d)	331 ± 105	357 ± 144	0.407	0.194
	Proteins ^¥¥^ (g/d)	96.9 ± 27.1	94.4 ± 34.3	0.738	0.676
	Lipids ^¥¥^ (g/d)	91.8 ± 34.5	100 ± 47.9	0.422	0.054
	Saturated FA ^¥¥^ (%)	25.7 ± 5.19	25.6 ± 4.59	0.927	0.108
	Monounsaturated FA ^¥¥^ (%)	46.3 ± 10.5	47.4 ± 9.64	0.683	0.478
	Polyunsaturated FA (%)	28.0 ± 13.7	27.1 ± 8.82	0.749	0.189
	Cholesterol ^¥¥^ (mg/d)	297 (146–384)	331 (97.5–473)	0.980	0.314
	Fibres ^¥¥^ (g/d)	24.6 ± 8.48	21.3 ± 8.01	0.109	0.844
	Magnesium (mg/d)	224 ± 86.2	176 ± 83.4	0.024	0.102

Values presented in the table are expressed as mean ± standard deviation. Kg: kilograms; HbA1c: glycated hemoglobin; HDL: high-density lipoprotein. ^¥^, adjusted for type of diabetes, age, and BMI. ^¥¥^, adjusted for type of diabetes, age, BMI, cholesterol, and HbA1c.

**Table 2:** Comparison of Demographic and Biochemical Parameters by Sweet Taste Sensitivity.

		Low-Sweet Tasters *n* = 54	High-Sweet Tasters *n* = 12	*p*	*p*
Gestational diabetes (*n*)		5 (15.6)	28 (82.4)		
Diabetes before pregnancy (*n*)		27 (84.4)	6 (17.6)	<0.001	
Taste thresholds for sucrose (Mmol/L) ^¥¥^		0	0.50 (0.25–1.00)	<0.001	<0.001
BMI (Kg/m^2^)		30.2 ± 5.88	32.0 ± 4.81	0.320	
Weight (kg)		77.8 ± 15.7	80.1 ± 12.7	0.637	
Laboratory tests	Fasting Glycemia ^¥^ (mmol/L)	6.90 ± 3.01	6.00 ± 1.35	0.118	0.192
	HbA1c ^¥^ (%)	6.84 ± 1.73	5.82 ± 0.56	0.001	0.461
	Cholesterol ^¥^ (mmol/L)	5.04 ± 1.03	6.15 ± 1.28	0.002	0.093
	HDL (mmol/L)	1.41 ± 0.25	1.55 ± 0.42	0.131	0.769
	Triglycerides ^¥^ (mmol/L)	1.31 (1.02–1.69)	1.75 (1.57–2.32)	0.016	0.336
Dietary survey	Calories ^¥¥^ (Kcal/d)	2579 ± 889	2817 ± 1011	0.416	0.166
	Carbohydrates ^¥¥^ (g/d)	336 ± 131	382 ± 103	0.257	0.157
	Proteins ^¥¥^ (g/d)	95.4 ± 31.1	96.4 ± 30.4	0.922	0.366
	Lipids ^¥¥^ (g/d)	95.2 ± 37.4	100 ± 59.7	0.783	0.277
	Satured fatty acid ^¥¥^ (%)	25.6 ± 4.63	26.1 ± 5.99	0.726	0.442
	Monounsattured FA ^¥¥^ (%)	46.3 ± 10.3	49.3 ± 8.42	0.353	0.709
	Polyunsaturated FA (%)	28.2 ± 11.8	24.6 ± 9.29	0.328	0.985
	Dietary cholesterol (mg/d)	311 (144–396)	191 (76.0–579)	0.777	0.851
	Fibers (g/d)	22.3 ± 8.34	25.6 ± 8.13	0.211	0.004
	Magnesium (mg/d)	199 ± 89.0	203 ± 84.6	0.882	0.300
	Vitamin C (mg/d)	91.0 (47.0–144)	141 (61.0–230)	0.100	0.004

Kg: kilograms; HbA1c: glycated hemoglobin; HDL: high-density lipoprotein; LDL: low-density lipoproteins. ^¥^, adjusted for type of diabetes, age, and BMI. ^¥¥^, adjusted for type of diabetes, age, BMI, cholesterol, and HbA1c.

## Data Availability

Data are available in this link: https://doi.org/10.6084/m9.figshare.29669672.
